# Survey of actual conditions of erythema marginatum as a prodromal symptom in Japanese patients with hereditary angioedema

**DOI:** 10.1016/j.waojou.2021.100511

**Published:** 2021-02-06

**Authors:** Isao Ohsawa, Atsushi Fukunaga, Shinya Imamura, Kazumasa Iwamoto, Akio Tanaka, Michihiro Hide, Daisuke Honda, Kouhei Yamashita, Chisako Fujiwara, Osamu Ishikawa, Takeo Yamaguchi, Junichi Maehara, Tomoya Hirose, Masahiro Ieko, Kunihiko Umekita, Yuya Nakamura, Hiromichi Gotoh

**Affiliations:** a Department of Nephrology, Internal Medicine, Saiyu Soka Hospital, Soka City, Saitama, Japan; b Division of Dermatology, Department of Internal Related, Kobe University Graduate School of Medicine, Kobe, Hyogo, Japan; c Department of Dermatology, Graduate School of Biomedical and Health Sciences, Hiroshima University, Hiroshima City, Hiroshima, Japan; d Department of Nephrology, Juntendo University Faculty of Medicine, Bunkyo-ku, Tokyo, Japan; e Department of Hematology and Oncology, Graduate School of Medicine, Kyoto University, Sakyo-ku, Kyoto, Japan; f Department of Dermatology, Gunma University Graduate School of Medicine, Maebashi City, Gunma, Japan; g Department of Gastroenterology, Japanese Red Cross Nagoya Daiichi Hospital, Nagoya City, Nagoya, Japan; h Department of Acute Care and General Medicine, Saiseikai Kumamoto Hospital, Kumamoto City, Kumamoto Japan; i Department of Traumatology and Acute Critical Medicine, Osaka University Graduate School of Medicine, Suita City, Osaka Japan; j Emergency and Critical Care Medical Center, Osaka Police Hospital, Osaka City, Osaka, Japan; k Department of Internal Medicine, School of Dentistry, Health Sciences University of Hokkaido, Ishikari-Tobetsu, Hokkaido, Japan; l Department of Rheumatology, Infectious Diseases and Laboratory Medicine, University of Miyazaki, Miyazaki City, Miyazaki, Japan

**Keywords:** Hereditary angioedema, Prodromal symptom, Erythema marginatum, Bradykinin, C1-inhibitor, Icatibant, ANGPT1, angiopoietin 1, BKB2-A, bradykinin-B2-receptor antagonist, C1–INH, C1-inhibitor, EM, erythema marginatum, F12, factor XII, HAE, hereditary angioedema, HAEnC1-INH, HAE with normal C1-inhibi tor, HAE-1/2, HAE types I and II, KNG1, kininogen 1, pdC1-INH, plasma derived- C1INH, PLG, plasminogen, SERPING1, serpin family G member 1

## Abstract

**Background:**

Hereditary angioedema (HAE) is a rare but life-threatening condition. HAE types I and II (HAE-1/2) result from C1-inhibitor (C1–INH) deficiency. However, recent genetic analysis has established a new type of HAE with normal C1–INH (HAEnC1-INH). The mutations of factor XII, plasminogen, angiopoietin 1, and kininogen 1 genes may be the cause of HAEnC1-INH. Nevertheless, other causative molecules (HAE-unknown) may be involved. The Japanese therapeutic environment for HAE has been improving owing to the self-subcutaneous injection of icatibant, which was approved for the treatment of acute attack and enables early therapy. Erythema marginatum (EM) is a visible prodromal symptom which occasionally occurs prior to an angioedema attack; hence, recognizing the risk of an acute attack is important for early treatment. However, the detailed characteristics of EM remain unclear. In this study, we first investigated the clinical manifestations of EM in Japanese patients with HAE.

**Methods:**

A 20-point survey was developed and distributed to 40 physicians to gather clinical data on EM from patients with HAE.

**Results:**

Data on 68 patients with HAE (58 patients with HAE-1/2 and 10 patients with HAE-unknown) were collected. Of the patients with HAE-1/2, 53.4% experienced EM, whereas 43.1% did not. The forearm was the most frequent area of EM (64.5%), followed by the abdomen (29.0%) and upper arm and precordium (19.3%). Of the HAE-1/2 patients with EM, 41.9% always had angioedema following EM, while 29.0% always had colocalization of EM with angioedema. Moreover, 3.2% showed a correlation between the awareness of EM and severity of an angioedema attack. In 60.9% of HAE-1/2 patients with EM, the interval between the awareness of EM and appearance of angioedema was <3 h. Of the patients with HAE-unknown, 30.0% also experienced EM.

**Conclusion:**

We confirmed that more than one-half of Japanese patients with HAE-1/2 and one-third of those with HAE-unknown develop EM as the prodromal symptom of an angioedema attack. Physicians should communicate the significance of EM to patients with HAE to prepare them for possible imminent attacks.

## Introduction

Hereditary angioedema (HAE) types I and II (HAE-1/2) refer to an autosomal dominant and potentially life-threatening disease caused by mutations of the gene “serpin family G member 1” (*SERPING1*) encoding the C1-inhibitor (C1–INH). Low C1–INH activity leads to excessive local formation of bradykinin, which causes dilatation and increases the permeability of vessels, via activation of the factor XII-driven plasma contact system.^1^^,^^2^ In Japan, plasma-derived C1–INH (pdC1-INH) has been used for the treatment of acute attacks of HAE. However, the injection of pdC1-INH is performed by medical professionals at medical facilities and self-injection has not been approved.^3^ At the end of 2018, the self-subcutaneous injection of icatibant (bradykinin-B2-receptor antagonist [BKB2-A]), which enables early therapy, was approved for the treatment of acute attacks. This development has offered benefits to patients with HAE in terms of quality of life. Notably, recent research revealed that HAE occurs in patients without low C1–INH activity, and this condition was termed HAE with normal C1-inhibitor (HAEnC1-INH). Some principal mutations were identified in the molecules of factor XII (F12), plasminogen (PLG), angiopoietin 1 (ANGPT1), and kininogen 1 (KNG1) genes.^4^, ^5^, ^6^, ^7^ Furthermore, the patients with HAE without previously reported gene mutations were categorized (HAE-unknown).^8^ In patients with HAEnC1-INH, we are groping for an adequate therapeutic choice.

Early treatment is key in the context of any therapeutic approach. Nevertheless, patients with HAE often do not receive treatment in the early phase of an attack, as they are unable to discriminate whether a symptom is derived from a real attack. Therefore, the prodromal symptoms of an angioedema attack have recently received considerable attention. Erythema marginatum (EM), malaise, irritability, anxiety, fatigue, and nausea are representative prodromal symptoms.^9^^,^^10^ Reshef et al reported that 82–95% of patients with HAE-1/2 develop these prodromal symptoms.^11^ Above all, EM is a visible prodromal symptom, and it has been shown that 40–60% of Caucasian patients with HAE-1/2 experience EM.^9^^,^^12^^,^^13^ However, the characteristics of EM have not been examined in depth. Hence, we performed this retrospective survey regarding EM in Japanese patients with HAE.

## Materials and methods

**Fig. 1:**
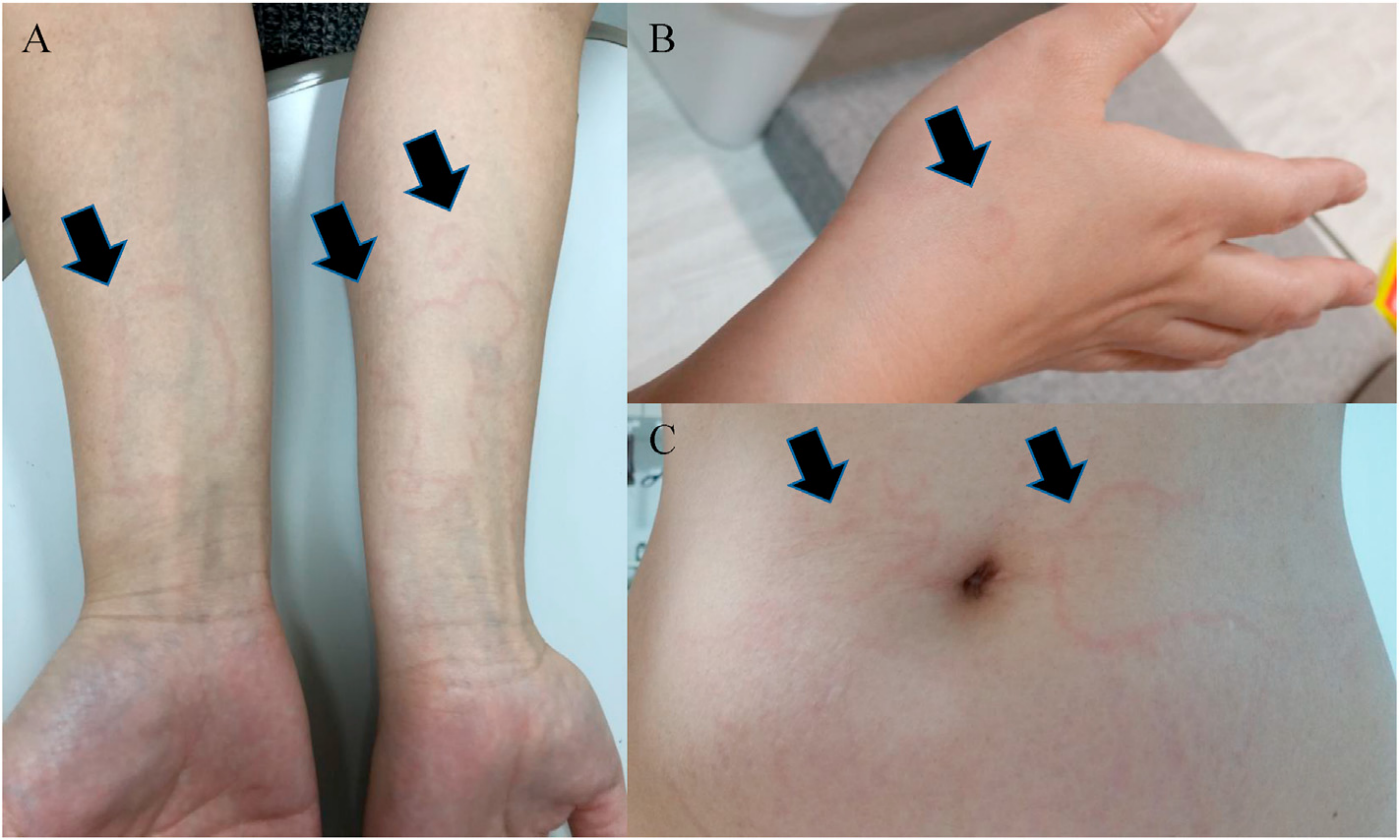
Representative images of erythema marginatum shown to a patient with HAE during the interview survey. (A) Closed winded lines on bilateral forearms. (B) Type “C”-like red line on the right dorsum of the hand. (C) Map-like fringe red lines on the abdomen. HAE, hereditary angioedema

A survey was conducted between June 2019 and May 2020 in Japan. The questionnaire was distributed to 40 physicians who participated in the Japanese network group “HAE FORUM”. All physicians who had experience in the diagnosis and treatment of patients with HAE in their daily practice were requested to participate in this study. Physicians showed representative photographs of EM (Fig. 1) to patients with HAE and asked questions to gather clinical data. All physicians provided written informed consent for their participation in this study. All involved personal information of the patients was removed, and the use of some numbers was not associated with personal identity. The procedure was approved by the institutional Ethics Committee.

The 20 questions (Supplementary Table 1) included age at the date of the survey, sex, HAE type, serum level of C4 and C1–INH activity at the time of diagnosis, and family history of angioedema. We asked whether the patients had a history of EM. For patients with a history of EM, the survey was continued with questions on the following parameters: location; sensation (itching or tingling); interval between the awareness of EM and appearance of angioedema; location of angioedema; and correlation between EM and self-assessment of the severity of angioedema. Regarding the most recent angioedema attack, the choice of therapies was investigated. Furthermore, the intervals between the disappearance of EM or angioedema and treatment were determined. The results of the genetic analysis of SERPING 1, F12, PLG, ANGP1, and KNG1 were reinvestigated for all cases classified as HAEnC1-INH.

## Results

Responses were received from 13 of the 40 physicians (32.5%). Data were collected from 58 patients with HAE-1/2 (19 males and 39 females) and 10 patients with HAE-unknown (2 males and 8 females).

### Results from patients with HAE-1/2

The characteristics of the patient population are presented in Table 1. The mean age was 45.3 years (range: 15–81 years). The estimated mean time from the onset of initial symptoms to diagnosis was 18.8 years (range: 0–60 years). The frequency distribution of angioedema attack was as follows: 0 times/year, 4 (6.9%); 1–5 times/year, 19 (32.8%); 6–10 times/year, 13 (22.4%); 11–15 times/year, 7 (12.1%); 16–20 times/year, 6 (10.3%); 21–25 times/year, 1 (1.7%); 26–30 times/year, 3 (5.2%); 41–45 times/year, 1 (1.7%); and >50 times/year, 3 (5.2%). A family history of angioedema was recorded in 48 patients (82.8%), whereas 9 patients (15.5%) did not have a positive family history; there were no available clinical data on family history for remaining 1 patient. Of the 58 patients, 31 (53.4%) had experienced EM.

**Table 1:** Characteristics of 58 patients with hereditary angioedema types I and II with C1-inhibitor deficiency

Sex, number (%)	
Male	19 (32.8)
Female	39 (67.2)
Mean age, years (range)	
At data abstraction	45.3 (15–81)
Time from onset of initial symptoms to diagnosis, mean years (range)	18.8 (0–60)
Frequency distribution of angioedema attack, times/year, number (%)	
0	4 (6.9)
1–5	19 (32.8)
6–10	13 (22.4)
11–15	7 (12.1)
16–20	6 (10.3)
21–25	1 (1.7)
26–30	3 (5.2)
31–35	0 (0.0)
36–40	0 (0.0)
41–45	1 (1.7)
46–50	0 (0.0)
>50	3 (5.2)
Family history of angioedema, number (%)	
Yes	48 (82.8)
No	9 (15.5)
No description	1 (1.7)
History of erythema marginatum, number (%)	
Yes	31 (53.4)
No	25 (43.1)
Unsure	2 (3.4)

The details of EM are summarized in Table 2. Of the 31 patients, EM with itching and tingling sensations was observed in 7 (22.6%) and 6 (19.3%) patients, respectively. Most of the patients (71.0%) did not have a sensation of itching or tingling. The location of EM in each patient was determined. The most common part of the body in which EM occurred was the forearm (64.5%), followed by the abdomen (29.0%), and the upper arm and precordium (19.3%). The occurrence of angioedema after EM was evaluated. Of the 31 patients, 13 (41.9%) and 18 (58.1%) always and occasionally experienced angioedema after the occurrence of EM, respectively. Colocalization of EM and angioedema was observed always in 9 patients (29.0%), occasionally in 16 patients (51.6%), and never in 6 patients (19.4%). A correlation between the awareness of EM and severity of angioedema was observed always in 1 patient (3.2%), never in 22 patients (71.0%), and was unknown in 8 patients (25.8%). Of the 31 patients, 24 provided information on the intervals between the awareness of EM and appearance of angioedema (Table 3). The intervals demonstrated a wide range (0–48 h), and 14 of 23 patients (60.9%) reported an interval <3 h.

**Table 2:** Details of erythema marginatum in 31 patients with hereditary angioedema types I and II

Erythema marginatum with:	
Itching sensation, number (%)	
Yes	7 (22.6)
No	22 (71.0)
Unknown	2 (6.4)
Tingling sensation, number (%)	
Yes	6 (19.3)
No	22 (71.0)
Unknown	3 (9.7)
Total number of patients per location of erythema marginatum, number (%)	
Upper arm	6 (19.3)
Elbow	3 (9.7)
Forearm	20 (64.5)
Dorsum of hand	4 (12.9)
Finger	1 (3.2)
Palm	1 (3.2)
Precordium	6 (19.3)
Back	2 (6.5)
Abdomen	9 (29.0)
Femur	5 (16.1)
Knee	1 (3.2)
Lower leg	3 (9.7)
Ankle	1 (3.2)
Dorsum of foot	2 (6.5)
Sole of foot	1 (3.2)
Angioedema occurring after erythema marginatum, n (%)	
Always	13 (41.9)
Sometimes	18 (58.1)
Colocation of erythema marginatum and angioedema, number (%)	
Always	9 (29.0)
Sometimes	16 (51.6)
Never	6 (19.4)
Relationship between awareness of erythema marginatum and severity of angioedema, number (%)	
Always	1 (3.2)
Never	22 (71.0)
Unknown	8 (25.8)

**Table 3:**
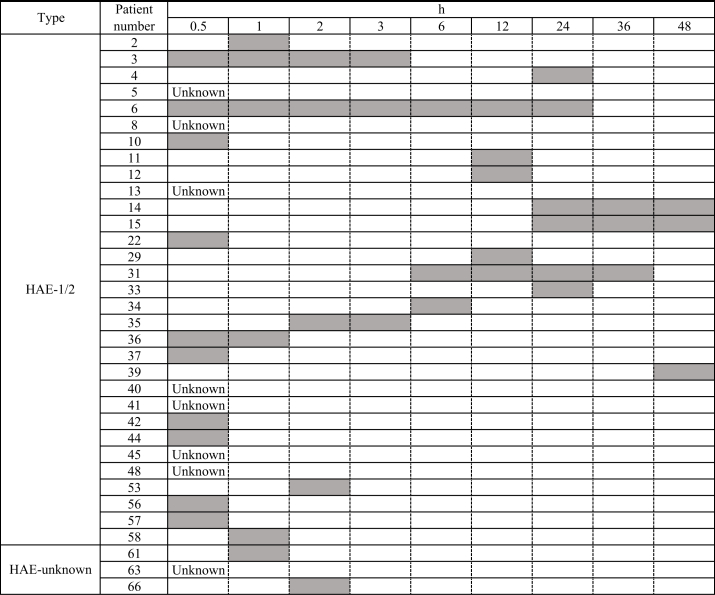
Variations in the interval between the awareness of erythema marginatum and appearance of angioedema in each HAE patient with C1 inhibitor deficiency and HAE-unknown. Abbreviations: HAE-1/2, hereditary angioedema types I and II; HAE-unknown, hereditary angioedema without previously reported gene mutatio Gray bar denotes the variations in the intervals between the awareness of erythema marginatum and appearance of angioedema

Of the 31 patients, 13 provided details on the most recent angioedema attack with EM (Table 4). Six patients (patient No. 2, 31, 35, 39, 57, 58) had angioedema after EM but did not receive treatment. Seven patients (patient No. 5, 10, 12, 22, 29, 42, 53) had received BKB2-A or pdC1-INH after the occurrence of angioedema. In all cases, EM disappeared prior to the disappearance of angioedema. EM disappeared following treatment in 5 of 6 patients (patient No. 5, 10, 22, 42, 53). Only in 1 patient (No. 12), EM spontaneously disappeared prior to treatment with BKB2-A.

**Table 4:** The most recent angioedema attack with erythema marginatum in HAE-1/2 and HAE-unknown.

Type	Patient number	Interval between the appearance of erythema marginatum and angioedema (h)	Treatment	Interval between the treatment and disappearance of erythema marginatum (h)	Interval between the treatment and disappearance of angioedema (h)
HAE-1/2	2	1	None	–	–
	5	Unknown	BKB2-A	1	10
	10	0	pdC1-INH	24	48
	12	72	BKB2-A	−24	0.6
	22	0	BKB2-A	24	24
	29	12	BKB2-A	Unknown	Unknown
	31	30	None	–	–
	35	2–3	None	–	–
	39	48	None	–	–
	42	0.1	pdC1-INH	12	24
	53	5	BKB2-A	6	12
	57	0.1	None	–	–
	58	1	None	–	–
HAE-unknown	61	Unknown	pdC1-INH	1	1
	63	24	BKB2-A	1	48
	66	2	pdC1-INH	Unknown	1

Abbreviations: HAE-1/2, hereditary angioedema types I and II; HAE-unknown, hereditary angioedema without previously reported gene mutations; BKB2-A, bradykinin-B2-receptor antagonist; pdC1-INH, plasma derived C1-inhibitor

### Results from patients with HAE-unknown

Ten patients assigned to HAEnC1-INH did not have previously detected gene mutations, such as *SERPING1*, *F12*, *ANGPT1*, *PLG*, and *KNG1*.^1^^,^^4^, ^5^, ^6^, ^7^ These patients had a family history of angioedema; thus, they were subclassified as HAE-unknown (Supplementary Table 2). The mean age was 26.9 years (range: 6–48 years). The estimated mean time from the onset of initial symptoms to diagnosis was 10.6 years (range: 3–35 years). Three of the 10 patients (30.0%; patient No. 61, 63, and 66) had experienced EM. Patient No. 61 (34 years old, female) did not have a sensation around the area of EM (located at the dorsum of the foot), and always developed angioedema after the occurrence of EM. Patient No. 63 (9 years old, female) did not have a sensation around the area of EM (located at the cheek, back, and femur), and occasionally developed angioedema after the occurrence of EM. Patient No. 66 (6 years old, female) had itching and tingling around the area of EM (located at the crown, cheek, dorsum of the hand, and lower leg), and always developed angioedema after the occurrence of EM. A correlation between the appearance of EM and severity of angioedema was always observed in patient No. 61; in others, this correlation remained unknown. The interval between the appearance of EM and angioedema was within 1 and 2 h for patients No. 61 and No. 63, respectively (Table 3). The 3 aforementioned patients received treatment (pdC1-INH or BKB2-A) for the most recent angioedema attack (Table 4). The EM in patients No. 61 and 63 disappeared within 1 h after treatment.

## Discussion

Although most prodromal symptoms are vague, EM is an objective symptom. Awareness regarding EM may be useful for recognizing the risk of an imminent full-scale attack of angioedema. In this study, we confirmed that 53.4% of Japanese patients with HAE-1/2 had experienced EM. According to Hungarian and Danish cohort studies, 42.0% (29/69) and 56.3% (49/87) of patients experienced EM, respectively.^12^^,^^14^ Therefore, we may infer that the incidence of EM was similar between Japanese and Caucasian patients with HAE-1/2.

EM is often observed in patients with rheumatic fever and psittacosis. Autoimmune mechanisms may be involved in the pathophysiology of these 2 diseases, although the available evidence is not fully conclusive.^15^^,^^16^ In recent research on HAE, data from 2 studies clearly revealed that the mechanism of EM formation is strongly related to the generation of bradykinin after activation of the contact system, kallikrein-kinin system, and fibrinolysis system. Nguyen et al reported an enhanced plasma kallikrein activity during EM, which suggests the generation of bradykinin prior to an acute attack.^17^ Kőhalmi et al reported an elevation of the level of d-dimer during EM.^18^ However, our data demonstrated that EM did not accompany angioedema in all cases, and the severity of the subsequent attack was not necessarily correlated with the appearance of EM. Furthermore, more than half of the patients with HAE-1/2 did not always experience colocalized EM with angioedema. Although the mechanisms of EM and angioedema generation remain elusive, Starr et al reported consistent results in an observational study of a large family with HAE-1/2.^19^ Therefore, EM may suggest the initiation of excess formation of bradykinin in the superficial skin prior to the occurrence of angioedema.

To our knowledge, there were no previous reports investigating the locations of EM, and the interval between the awareness of EM and appearance of angioedema. Of course, some patients with HAE may be completely unaware of the risk of EM until they are properly informed by physicians. Although our data revealed that the actual distribution of EM may be wide, patients with HAE should regularly check their forearms, abdomen, precordium, and femur. On the other hand, the observed intervals between the appearance of EM and angioedema ranged 0–48 h. The activity of bradykinin-cleaving enzymes, such as neutral endopeptidase, angiotensin-converting enzyme, carboxypeptidase N, dipeptidyl peptidase 4, aminopeptidase 1, and aminopeptidase 2 differs between individuals.^20^ Hence, we hypothesized that there may be a link between differences in the activity of bradykinin-cleaving enzymes and disappearance of EM. Most cases of EM disappeared after treatment with BKB2-A or pdC1-INH, and before the disappearance of angioedema. Spontaneous disappearance of EM prior to treatment was recorded in one case (patient No. 12). If patients are aware of the appearance of EM several hours prior to the development of full-scale angioedema, they may have sufficient time to recognize a subsequent angioedema attack.

In Japan, there is very small number of HAEnC1-INH patients with identified gene mutations, and only two families with HAEnC1-INH have been reported as carrying a PLG gene mutation.^21^ None of our patients could be identified based on their gene mutations, and their diagnosis of HAE-unknown was reached through exclusion diagnosis. Regrettably, we could not gather data for >10 patients with HAE-unknown. There was a small number of patients who experienced EM, and we should keep in mind that the patients with HAEnC1-INH also experienced EM. A detailed assessment of the experience of EM prior to angioedema may provide a clue for the diagnosis of any type of HAE.

Compared with previous Japanese reports on HAE-1/2,^3^^,^^22^^,^^23^ patients with HAE-1/2 in this study exhibited similar characteristics, such as sex ratio, time from the onset of the initial symptom to diagnosis, frequency of angioedema, and a family history of angioedema. However, there were several limitations in this investigation. Our study included only 4 dermatologists, and the remaining physicians had another area of expertise. General practitioners have difficulty in discriminating EM from urticaria.^9^ There is possibility that the results regarding the characteristics of EM were affected by the frequency distribution of angioedema attack because >60% of patients with HAE-1/2 in this study experienced less than 1 attack per month. In addition, our survey did not include pediatricians, as there were no patients with HAE-1/2 aged <15 years. A previous report highlighted that EM could be observed even in newborns,^24^ and skin observations would be informative for early diagnosis. Regardless of patient age, further studies investigating the potential benefits of monitoring for prodromal symptoms are warranted.

In conclusion, EM is a valuable informative sign for recognizing the risk of a full-scale angioedema attack in Japanese patients with HAE. Physicians should inquire regarding prodromal symptoms in patients with HAE and offer advice on the usefulness of observing EM for the precognition of angioedema.

## Funding

Not applicable.

## Consent for publication

All authors have agreed with the content of the article, and agreed to submit to World Allergy Organization Journal.

## Author contributions

IO designed the study, interpreted the data, and drafted the manuscript. AF, SI, KI, AT, MH, DH, KY, CF, OI, TY, JM, TH, MI, and KU performed data collection and contributed equally to the review of the article. YN, and HG participated in the design of the study, and performed a critical review of the manuscript. All authors read and approved the final version of the manuscript.

Availability of data and material

All authors agree to share their raw data, any digital study materials, and analysis code as appropriate.

## Ethics approval

This study protocol was approved by the institutional review board (IRB) of Saiyu Soka Hospital (2019–007).

## Declaration of competing interest

The authors report no conflicts of interest.
