# Supplementary material for: Survey of actual conditions of erythema marginatum as a prodromal symptom in Japanese patients with hereditary angioedema
Source: World Allergy Organ J. 2021 Feb 6;14(2):100511. doi: 10.1016/j.waojou.2021.100511 (PMC7872976; doi:10.1016/j.waojou.2021.100511)
Supplement: Multimedia component 1 [file mmc1.docx]

| **Supplementary Table 1.** Erythema marginatum survey questions in Japanese patients with HAE | |
| --- | --- |
| Question | Response option |
| 1. What is the age of the patient at response? |  |
| 2. What is the sex of the patient? | Male |
|  | Female |
| 3. Which type of HAE has the patient been diagnosed with? | Type I |
|  | Type II |
|  | Type I or II |
|  | HAE with normal C1-inhibitor |
| 4. What was the level of C4 at diagnosis? (mg/dL) |  |
| 5. What was the level of C1-inhibitor activity at diagnosis? (%) |  |
| 6. How many days passed from the occurrence of the initial symptom to diagnosis? (years) |  |
| 7. How many attacks has the patient experienced in the past year? (times per year) |  |
| 8. Is there a family history of angioedema? | Yes |
|  | No |
|  | Unknown |
| 9. Has the patient ever experienced erythema marginatum? | Yes |
|  | No |
|  | Unknown |
| *If the patient answered 'Yes' to Question 9, the survey was continued with the following questions.* |  |
|  |  |
| 10. Does the patient have itching sensation? | Yes |
|  | No |
|  | Unknown |
| 11. Does the patient have tingling sensation? | Yes |
|  | No |
|  | Unknown |
| 12. Which location of erythema marginatum has the patient ever reported? |  |
| 13. Did angioedema occur after erythema marginatum? | Always |
|  | Sometimes |
| 14. Did erythema marginatum colocalize with angioedema? | Always |
|  | Sometimes |
|  | Never |
| 15. Is there an association between the appearance of erythema marginatum and severity of angioedema? | Always |
|  | Sometimes |
|  | Never |
| 16. What was the length of the interval between the awareness of erythema marginatum and appearance of angioedema? (h) |  |
|  |  |
| *If the most recent erythema marginatum occurred with angioedema, the following questions were continued.* |  |
|  |  |
| 17. What was the length of the interval between the awareness of erythema marginatum and appearance of angioedema? (h) |  |
| 18. Have you treated angioedema with any drugs? | None |
|  | Tranexamic acid |
|  | Anti-histamine |
|  | Corticosteroid |
|  | Plasma derived C1-inhibitor |
|  | Icatibant |
|  | Others |
| 19. What was the length of the interval needed until the disappearance of erythema marginatum after treatment? (h) |  |
| 20. What was the length of the interval needed until disappearance of angioedema after treatment? (h) |  |
|  |  |
| Abbreviations: HAE, hereditary angioedema |  |
