# Supplementary material for: Survey of actual conditions of erythema marginatum as a prodromal symptom in Japanese patients with hereditary angioedema
Source: World Allergy Organ J. 2021 Feb 6;14(2):100511. doi: 10.1016/j.waojou.2021.100511 (PMC7872976; doi:10.1016/j.waojou.2021.100511)
Supplement: Multimedia component 2 [file mmc2.docx]

**Supplementary Table 2.** Characteristics of 10 patients with HAE-unknown

| Sex, number (%) | |
| --- | --- |
| Male | 2 (20.0) |
| Female | 8 (80.0) |
| Mean age, years (range) | |
| At data abstraction | 26.9 (6–48) |
| Time from onset of initial symptoms to diagnosis*, years (range) | 10.6 (3–35) |
| Frequency distribution of angioedema attack*, times/year, number (%) | |
| 1–5 | 5 (50.0) |
| 6–10 | 2 (20.0) |
| >50 | 2 (20.0) |
| Family history of angioedema, number (%) |  |
| Yes | 10 (100.0) |
| No | 0 (0.0) |
| History of erythema marginatum, number (%) |  |
| Yes | 3 (30.0) |
| No | 7 (70.0) |

*: Data for one patient were deficient.

Abbreviations: HAE-unknown, hereditary angioedema without previously reported gene mutations
